# Supplementary material for: High-resolution promoter interaction analysis implicates genes involved in the activation of Type 3 Innate Lymphoid Cells in autoimmune disease risk
Source: bioRxiv. 2026 Jan 10:2022.10.19.512842. Preprint. [Version 4] doi: 10.1101/2022.10.19.512842 (PMC12821085; doi:10.1101/2022.10.19.512842)
Supplement: Supplement 15 [file NIHPP2022.10.19.512842v4-supplement-15.pdf]

bioRxiv preprint doi: https://doi.org/10.1101/2022.10.19.512842; this version posted January 11, 2026. The copyright holder for this preprint (which was not certified by peer review) is the author/funder, who has granted bioRxiv a license to display the preprint in perpetuity. It is made available under aCC-BY 4.0 International license.

Supplementary Figures

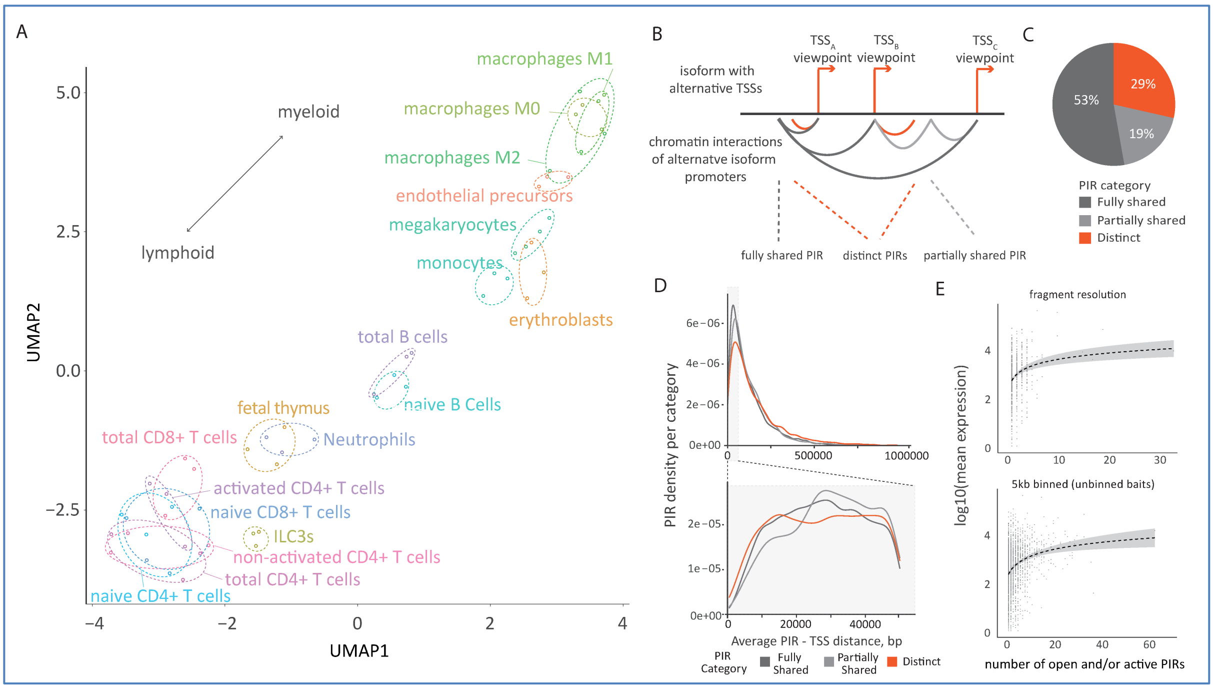

Figure S1. Compendium of promoter-enhancer interactions in ILC3s. A. UMAP of CHiCAGO scores detected for PCHi-C in ILC3s versus public data in 17 primary human blood cell types[30]. **B.** Scheme representing the classification of PIRs detected at alternative transcription start sites (ATSS) of the same gene: 'fully shared' (shared across all captured ATSSs), partially shared and distinct (unique to a single ATSS). **C.** Pie chart showing the degree of enhancer sharing across alternative transcription start sites (ATSS) for short-range contacts. **D.** Distance distribution of ATSS-specific and shared PIRs at 5kb binned (baits unbinned) resolution. Top panel - interactions up to 1Mb (Kruskal-Wallis test $p < 2.22e-16$; pairwise Wilcoxon test $p = 8.68e-6$ [partially shared vs fully shared], $p = 4.46e-8$ [partially shared vs distinct] and $p < 2.22e-16$ [fully shared vs distinct]; bottom panel - interactions up to 50kb (Kruskal-Wallis test $p = 7.65e-5$; pairwise Wilcoxon test $p = 9.8e-5$ [partially shared vs fully shared], $p = 6e-4$ [partially shared vs distinct] and $p = 1$ [fully shared vs distinct]). **E.** Correlation between gene expression and number of regulatory elements identified in CHiCAGO PIRs at fragment and 5kb (solitary baits) resolution.

bioRxiv preprint doi: https://doi.org/10.1101/2022.10.19.512842; this version posted January 11, 2026. The copyright holder for this preprint (which was not certified by peer review) is the author/funder, who has granted bioRxiv a license to display the preprint in perpetuity. It is made available under aCC-BY 4.0 International license.

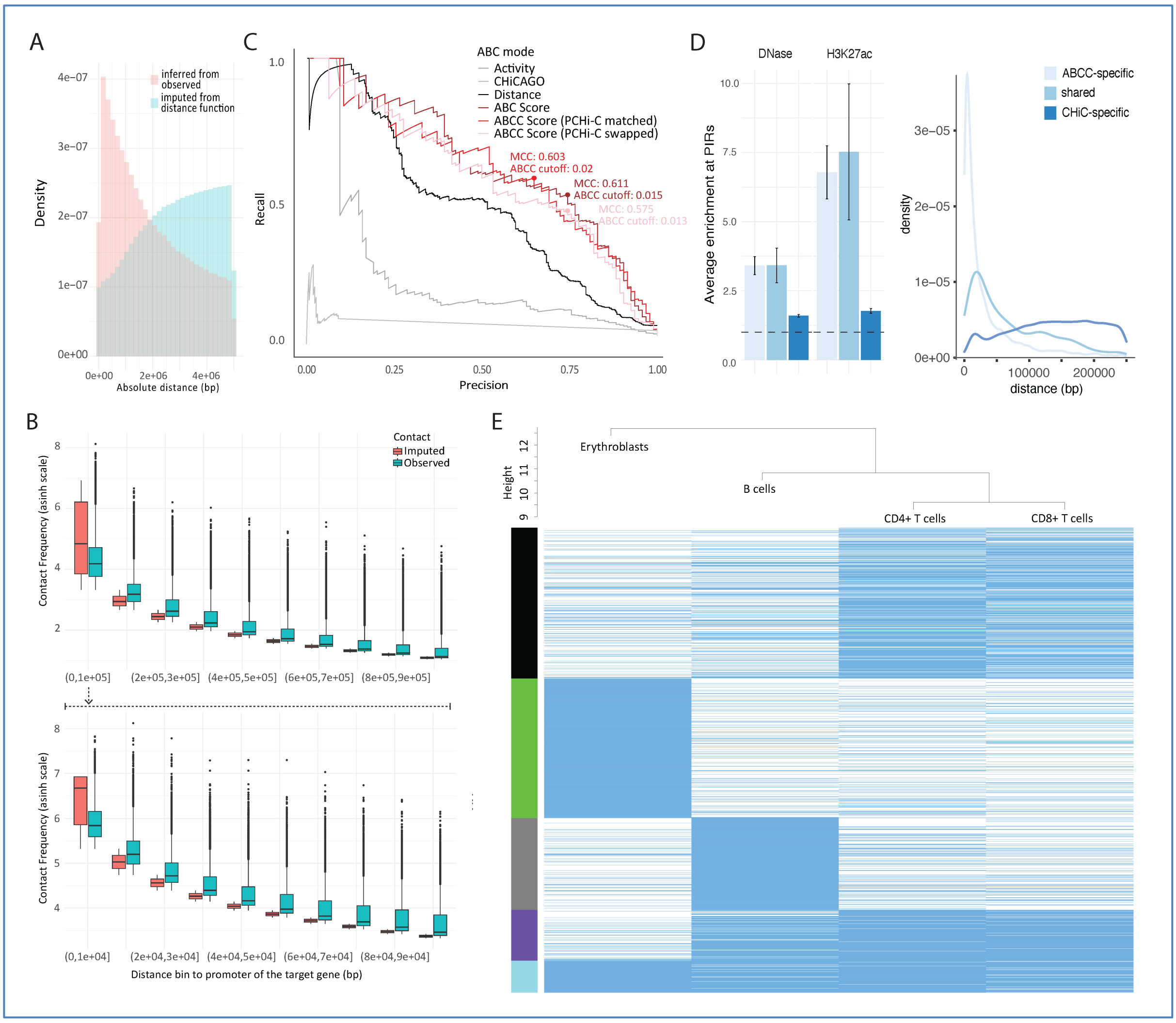

Figure S2. Benchmarking the ABCC approach with public data. A. Density distribution of promoter interactions inferred from observed PCHi-C contact frequencies (pink) and those imputed using the CHiCAGO distance function (cyan) across genomic interaction distances. **B.** Contact frequency distributions stratified by distance. Observed PCHi-C contacts are shown in green, imputed contacts (using expected frequencies estimated using the CHiCAGO distance function) are shown in blue. Similarly to standard ABC, frequency capping is introduced for short-range imputed contacts (<5kb). **C.** Precision–recall curves benchmarking the predictive performance of different scoring approaches for enhancer–promoter interactions in erythroblasts. Curves compare the scoring across: CHiCAGO-detected contacts, Activity alone, Distance alone, the conventional ABC score, and PCHi-C-based ABCC score in two modes: “matched” - using PCHi-C cell-type specific profile for erythroid cells and “swapped”, in which a PCHi-C dataset with a similar read coverage from a different cell type, CD4+ T cells, is used instead. MCC: Matthews correlation coefficient, an alternative to the AUC metric that is more informative under class imbalance and more sensitive to performance at a fixed decision threshold[175]. **D.** Enrichment of epigenetic markers at PIRs: DNase - chromatin accessibility and H3K27ac - active enhancers (left panel) and distance distribution of ABCC-specific, PCHiC-specific and shared enhancer-promoter links (right panel) in K562 cells for 0.023 ABCC threshold. **E.** Hierarchical clustering heatmap of enhancer–promoter interactions predicted with ABCC across cell types (erythroblasts, B cells, CD4+ T helper cells, CD8+ T cells).

bioRxiv preprint doi: https://doi.org/10.1101/2022.10.19.512842; this version posted January 11, 2026. The copyright holder for this preprint (which was not certified by peer review) is the author/funder, who has granted bioRxiv a license to display the preprint in perpetuity. It is made available under aCC-BY 4.0 International license.

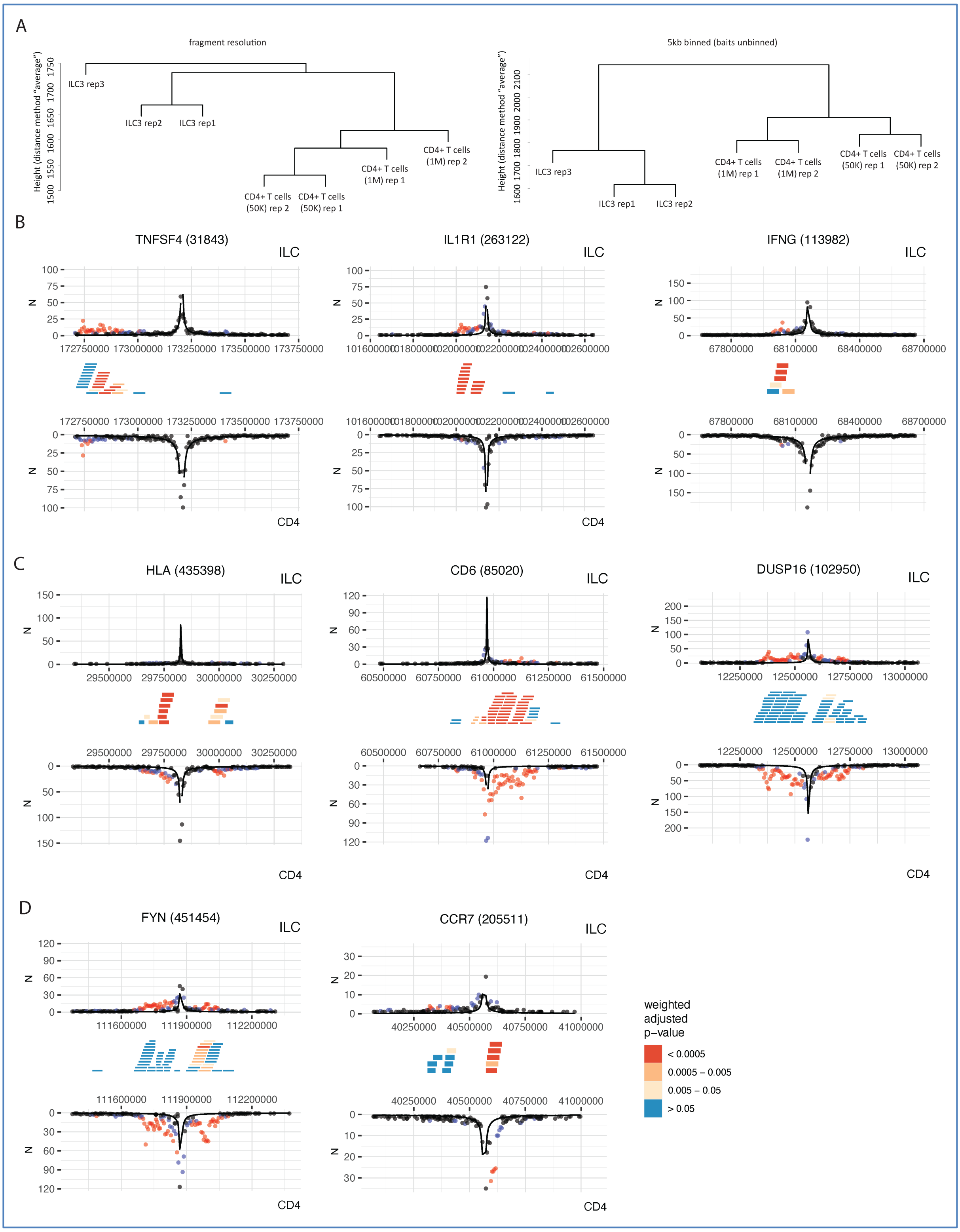

Figure S3. Genes with differential contacts in ILC3s and CD4+ T cells. A. Hierarchical clustering of ILC3s and CD4+ T cells PCHi-C datasets. **B-D.** Examples of captured promoters with differential wiring between ILC3s and CD4+ T cells: promoters with stronger **(B)** and weaker **(C)** contacts in ILC3s compared with CD4+ T cells, as well as with both types of contacts **(D).**

bioRxiv preprint doi: https://doi.org/10.1101/2022.10.19.512842; this version posted January 11, 2026. The copyright holder for this preprint (which was not certified by peer review) is the author/funder, who has granted bioRxiv a license to display the preprint in perpetuity. It is made available under aCC-BY 4.0 International license.

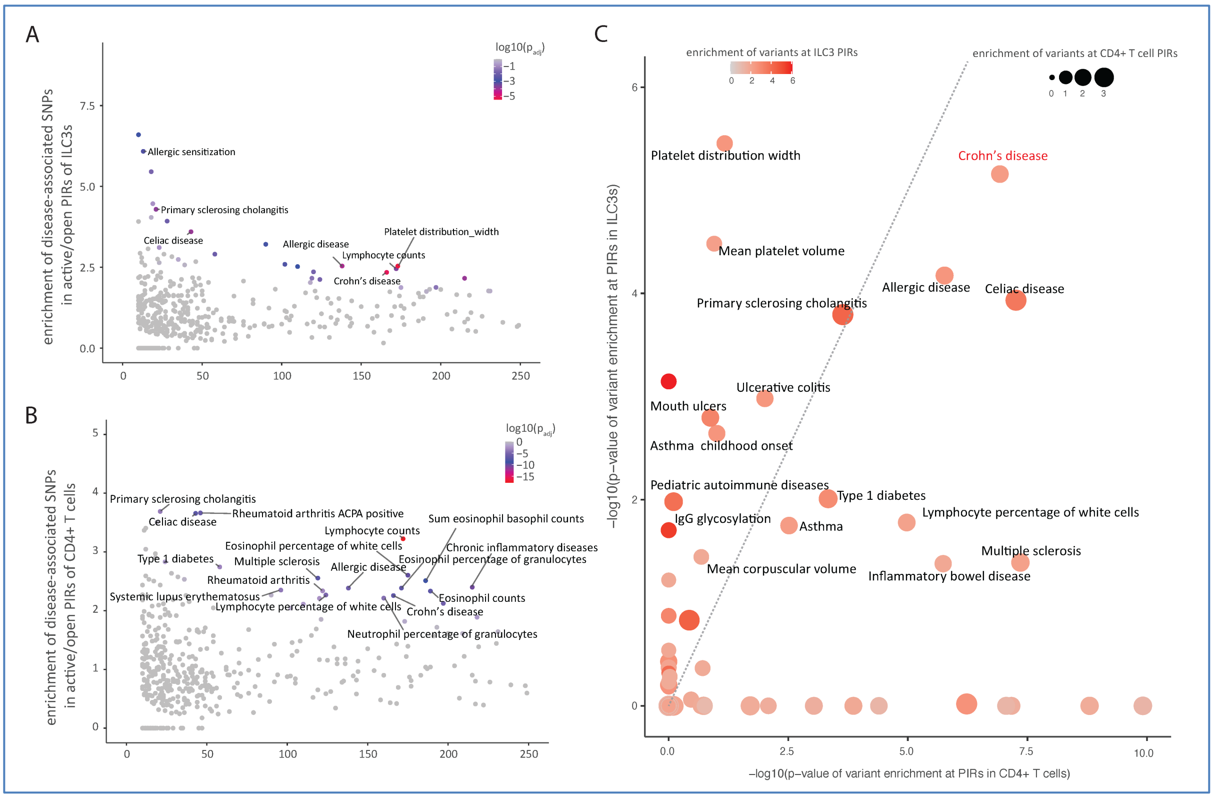

Figure S4. Supplementary information for the RELI analysis of risk loci enriched in ILC3 and CD4+ T-cell PIRs. A-B. RELI enrichment of risk variants in ILC3s **(A)** and CD4s PIRs (**B**) across 495 diseases and traits. Traits with log_{10}(BH corrected p-value in ILC3s) < 0.001, number of loci per trait > 10, and enrichment > 2.2 are labelled. **C.** Adjusted p-value of RELI enrichment of risk variants ILC3s vs CD4s PIRs across 495 diseases and traits. Traits with log_{10}(BH corrected p-value in ILC3s) < 0.05 are labelled.

bioRxiv preprint doi: https://doi.org/10.1101/2022.10.19.512842; this version posted January 11, 2026. The copyright holder for this preprint (which was not certified by peer review) is the author/funder, who has granted bioRxiv a license to display the preprint in perpetuity. It is made available under aCC-BY 4.0 International license.

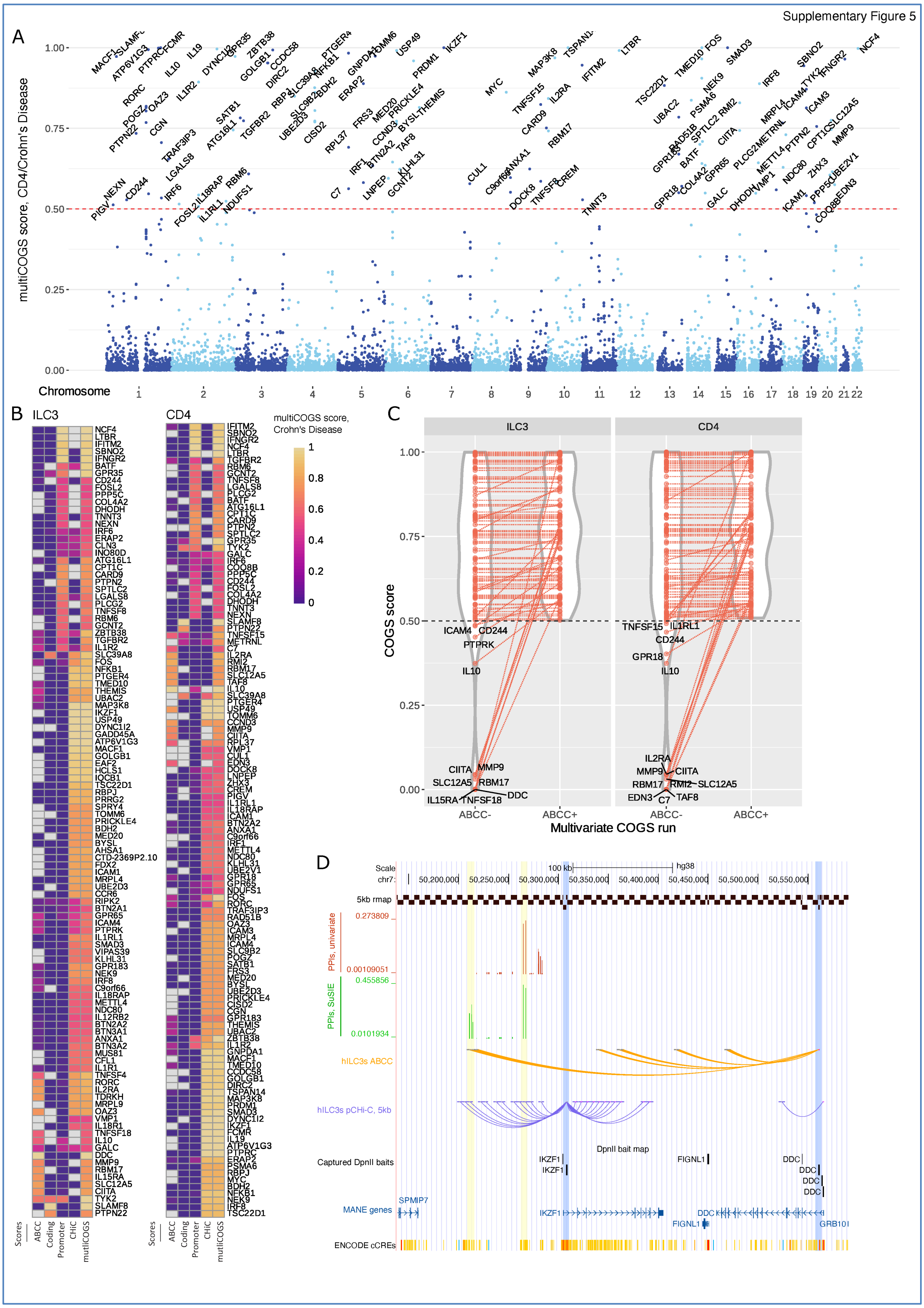

Figure S5. MultiCOGS prioritises gene sets in Crohn's Disease. A. Manhattan plot showing multiCOGS for CD risk based on promoter contacts in CD4+ T cells. **B.** Heatmaps of region contributions to multiCOGS scores in ILC3s and CD4s in CD. **C.** Illustration of genes that were only prioritised for CD with the addition of ABCC, in ILC3s and CD4s. In each graph, the multiCOGS score

bioRxiv preprint doi: https://doi.org/10.1101/2022.10.19.512842; this version posted January 11, 2026. The copyright holder for this preprint (which was not certified by peer review) is the author/funder, who has granted bioRxiv a license to display the preprint in perpetuity. It is made available under aCC-BY 4.0 International license.

with and without ABCC is plotted for all genes that were prioritised in the full multiCOGS run (score > 0.5 with ABCC). **D.** Illustration of multiCOGS prioritisation of *IKZF1* and *DDC* in ILC3s in the 7p locus. In this locus, multivariate fine mapping identifies two credible sets of variants (yellow bars), whereas univariate fine mapping only detects one. PCHi-C interactions connect these likely causal variants to the *IKZF1* promoter (first blue bar). However, ABCC interactions also connect one of the credible sets to the DDC promoter (second blue bar). Thus, multiCOGS prioritises both genes, whereas classic COGS prioritises only *IKZF1*.

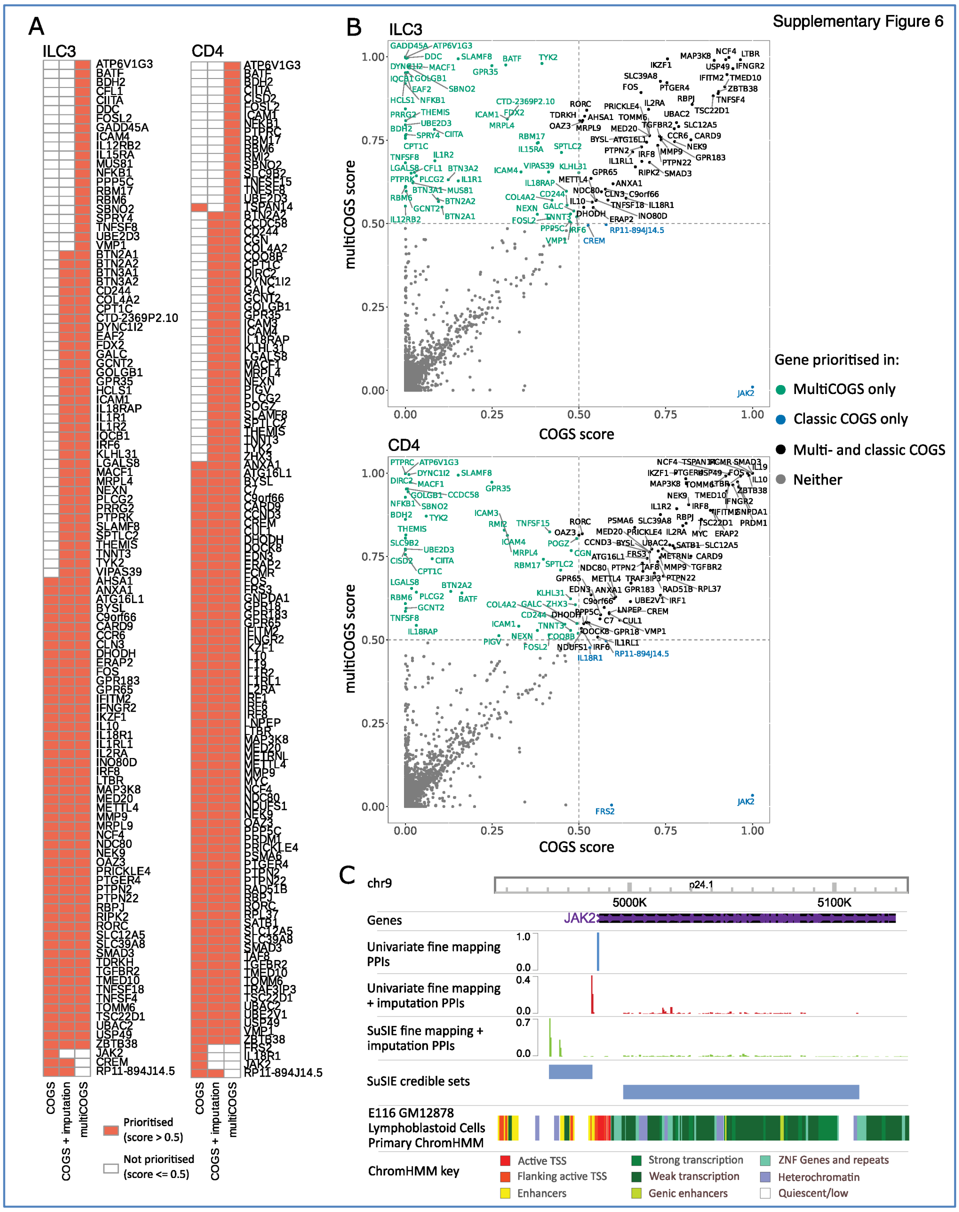

Figure S6. Comparison of gene prioritisation for Crohn's Disease in classic COGS versus multiCOGS. A. Comparison of prioritised gene sets between classic COGS, classic COGS plus imputation, and multiCOGS (i.e. imputation plus multivariate fine mapping, processed via the multiCOGS algorithm) for CD. Shown for ILC3s and CD4+ T cells. **B.** Comparison of COGS scores and multiCOGS scores for genes in ILC3 cells (top) and CD4+ T cells (bottom) for CD. Green labels indicate genes prioritised in multiCOGS only, blue in classic COGS only, and black in both. **C.** Plot of the *JAK2* locus, showing the shift of the most likely causal variant from the promoter of *JAK2* to a region around 20kb upstream of the promoter upon multivariate fine mapping, leading to a lower multiCOGS vs classic COGS score. No chromosomal interactions were observed between this region and the *JAK2* promoter in ILC3 or CD4+ T cells.

bioRxiv preprint doi: https://doi.org/10.1101/2022.10.19.512842; this version posted January 11, 2026. The copyright holder for this preprint (which was not certified by peer review) is the author/funder, who has granted bioRxiv a license to display the preprint in perpetuity. It is made available under aCC-BY 4.0 International license.

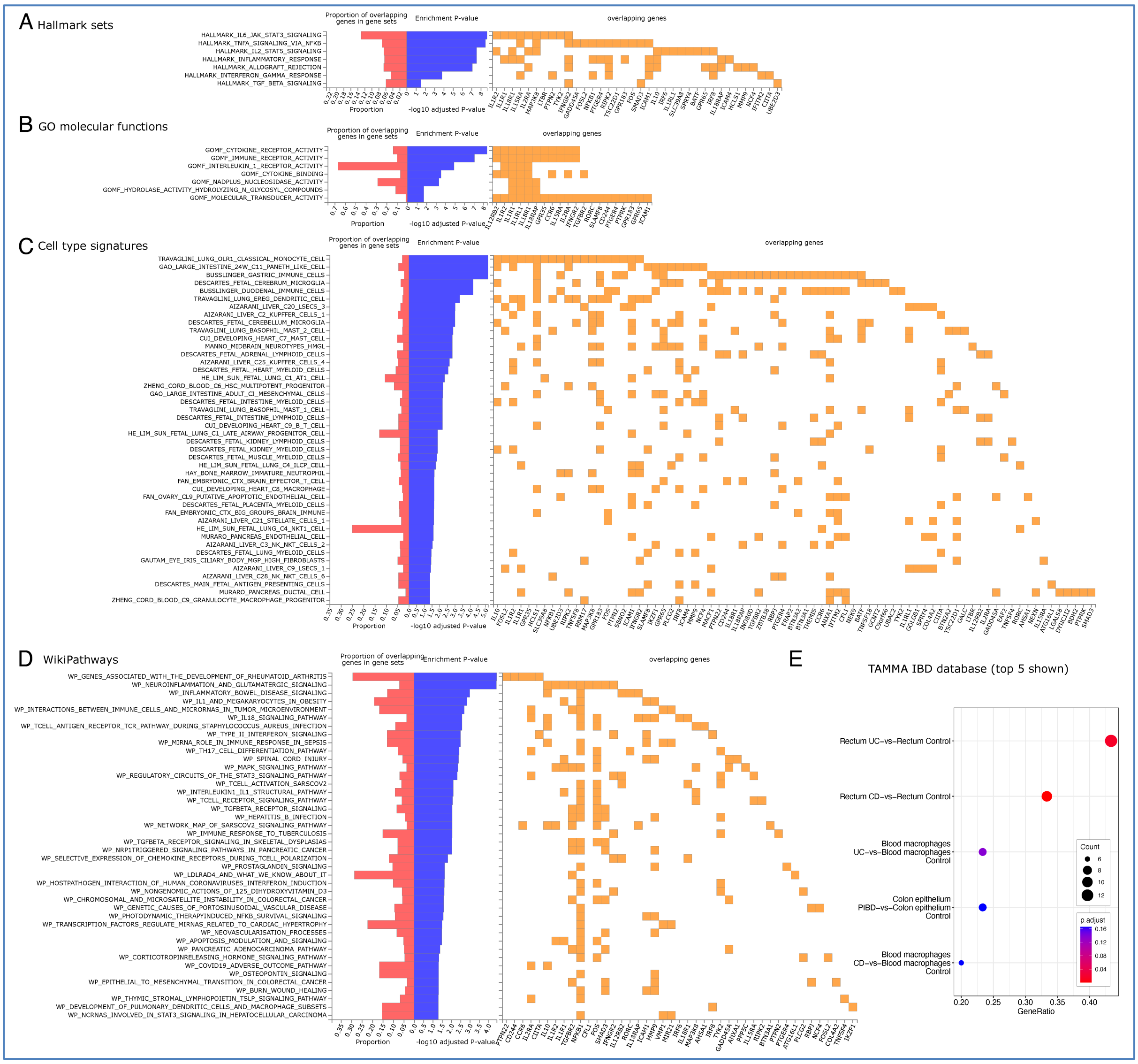

Figure S7. Biological annotation of multiCOGS CD genes in ILC3s. A-D. Enriched gene sets among multiCOGS genes detected using the GENE2FUNC pipeline in FUMA[121], for the following databases: (**A**) MSigDB hallmark sets, (**B**) GO molecular functions, (**C**) MSigDB cell type signatures, (**D**) MSigDB WikiPathways. **E.** Enrichment analysis for differentially expressed gene sets among multiCOGS genes in the TAMMA IBD database.

bioRxiv preprint doi: https://doi.org/10.1101/2022.10.19.512842; this version posted January 11, 2026. The copyright holder for this preprint (which was not certified by peer review) is the author/funder, who has granted bioRxiv a license to display the preprint in perpetuity. It is made available under aCC-BY 4.0 International license.

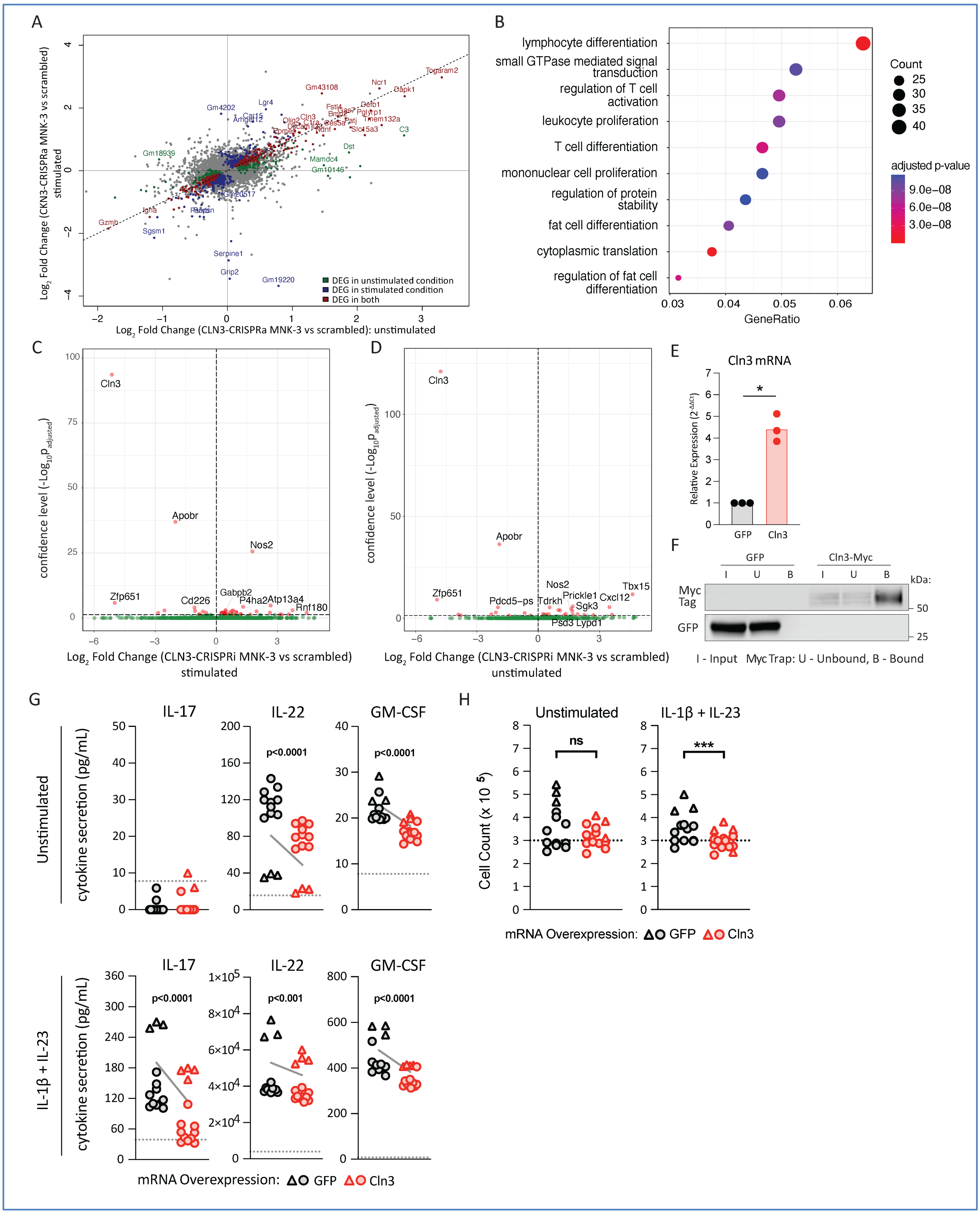

Figure S8. Additional information on the role of *Cln3* in ILC3 inflammatory function.
A. Comparison of differentially expressed genes in IL23/IL-1βl-stimulated vs unstimulated *Cln3*-CRISPRa cells (relative to scrambled gRNA controls). **B.** GO term enrichment analysis for genes differentially expressed upon CLN3-CRISPRa stimulation. **C.** Differential expression of genes in IL23/IL-1βl-stimulated *Cln3*-CRISPRi MNK-3 cells vs scrambled gRNA controls. Red - differentially expressed genes (DESeq2 adjusted p-value < 0.05), green - all other genes. **D.** Differential expression of genes in unstimulated *Cln3*-CRISPRi MNK-3 cells vs scrambled gRNA controls. Red - differentially expressed genes (DESeq2 adjusted p-value < 0.05), green - all other genes. **E.** *Cln3* expression in MNK-3 cells electroporated with Cln3-myc mRNA or GFP mRNA. Transcript abundance was quantified by qPCR,

bioRxiv preprint doi: https://doi.org/10.1101/2022.10.19.512842; this version posted January 11, 2026. The copyright holder for this preprint (which was not certified by peer review) is the author/funder, who has granted bioRxiv a license to display the preprint in perpetuity. It is made available under aCC-BY 4.0 International license.

normalised to *Hprt*, and expressed relative to the GFP mRNA control. Each point represents an independent experiment. Statistical significance was assessed using a paired Welch's t-test, $p<0.05$ (*). **F.** Verification of CLN3-myc protein expression and Myc tag-dependent pulldown. MNK-3 cells were lysed, subjected to immunoprecipitation using Myc-Trap agarose, and resolved by reducing SDS-PAGE. "I" = input lysate; "U" = unbound fraction; "B" = bead-bound fraction. Immunoblotting with anti-myc tag antibody detected a ~65–80 kDa Cln3-myc species selectively enriched in the bound fraction. **G.** Cytokine secretion upon *Cln3* overexpression across independent experiments. MNK-3 cells were electroporated with GFP mRNA (black) or Cln3-myc mRNA (red) and cultured for 24 hr under unstimulated (top row) or IL-1β + IL-23–stimulated (bottom row) conditions. Cytokine concentrations (IL-17, IL-22, GM-CSF) in culture supernatants were quantified by ELISA. Each symbol represents a biological replicate from two independent experiments (triangles vs circles). The dotted horizontal line indicates the lower limit of quantification for each assay. Statistical significance was assessed using a linear mixed-effects model with experiment as a random effect and transfection as a fixed effect (n=13–14 per condition). Solid grey lines indicate group means, with dotted grey bands indicating 95% confidence intervals of the fixed-effect. **H.** Cell numbers upon *Cln3* overexpression with and without inflammatory stimulation. MNK-3 cells were electroporated with GFP mRNA (black) or Cln3-myc mRNA (red) and cultured for 24 hr in unstimulated (left) or IL-1β + IL-23–stimulated (right) media. Each symbol represents a biological replicate from two independent experiments (triangles vs circles). Lines connect the experiment-specific means. Viable cell numbers were quantified by trypan blue exclusion. The dotted line indicates the number of cells seeded at 0 hr. Statistical significance was assessed using a linear mixed-effects model with experiment as a random effect and transfection as a fixed effect (n=13–14 per condition). Not significant (ns), $p<0.001$ (***).

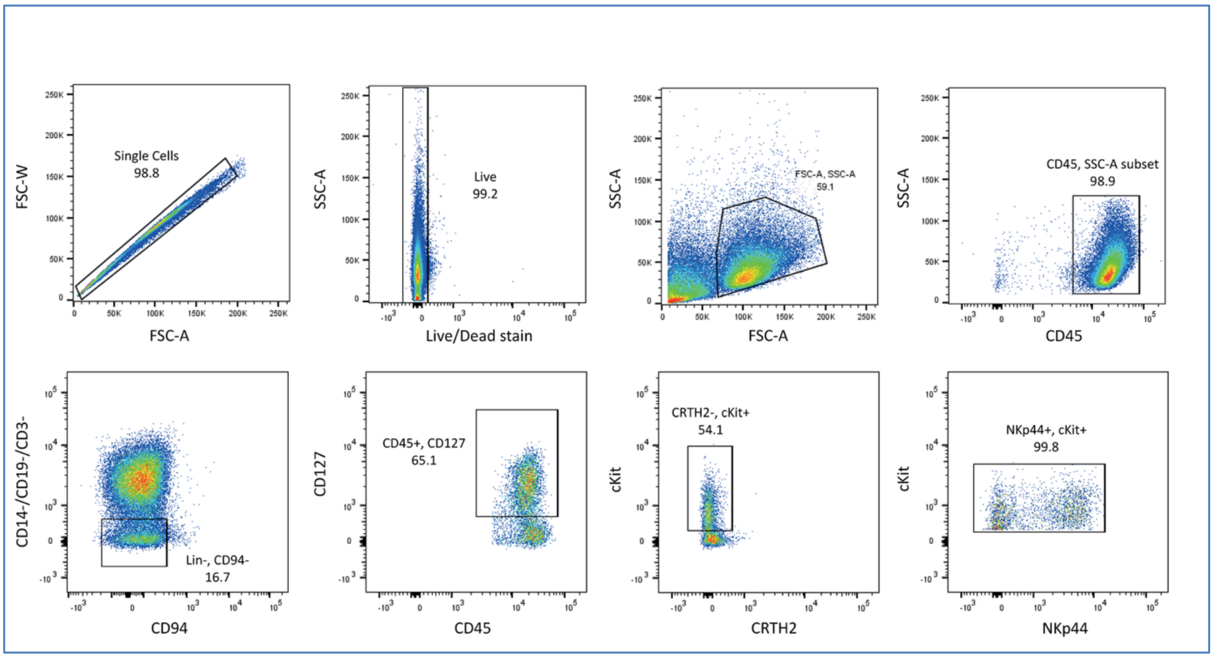

Figure S9. Flow cytometry gating strategy for isolation of human ILC3s from tonsils.

bioRxiv preprint doi: https://doi.org/10.1101/2022.10.19.512842; this version posted January 11, 2026. The copyright holder for this preprint (which was not certified by peer review) is the author/funder, who has granted bioRxiv a license to display the preprint in perpetuity. It is made available under aCC-BY 4.0 International license.

List of supplementary tables

Table S1. PCHi-C quality metrics.
Table S2. Pathway enrichment for genes with ILC3-specific PIRs
Table S3. Pathway enrichment for genes with CD4-specific PIRs
Table S4. Pathway enrichment for genes with differential PIRs between ILC3 and CD4+ T cells
Table S5. Pathway enrichment for genes with non-differential PIRs between ILC3 and CD4+ T cells
Table S6. RELI results
Table S7. Candidate genes prioritised by multiCOGS in ILC3 cells and CD4+ T cells for Crohn's Disease.
Table S8. Prior evidence for candidate genes prioritised by multiCOGS in ILC3 cells and CD4+ T cells for Crohn's Disease.
Table S9. Pathway enrichment for multiCOGS-prioritised CD candidate genes in ILC3s
Table S10. LOLA results for TF enrichment within the PIRs of CD genes in ILC3s
Table S11. Differentially expressed genes upon CRISPR perturbations targeted to the *Cln3* promoter in MNK-3 cells
Table S12. MultiCOGS results in ILC3 and CD4+ T cells across 6 autoimmune traits
Table S13. The biological functions of multiCOGS-prioritised genes across 6 autoimmune traits in ILC3s
Table S14. sgRNA and primer sequences for Cln3 CRISPR targeting

Supplementary Note 1.

MultiCOGS resulted in loss of five candidate genes in one or both cell types, compared with classic COGS (*JAK2*, *CREM*, *FRS2*, *IL18R1* and *RP11-894J14.5*; see **Fig. S6B**). Of these, we were intrigued by the loss of *JAK2* in both cell types, because it is a well-noted candidate gene in IBD, with JAK inhibitors already used to treat ulcerative colitis and CD[172]. The COGS score for *JAK2* was substantially lower across both cell types when genetic imputation and multivariate fine mapping were employed (classic COGS score ~1 in both cell types, multiCOGS score ~0.01 in ILC3s and ~0.03 in CD4s). Upon examining the locus, we discovered that fine mapping with the univariate methodology (Wakefield synthesis[173]) identified the most likely causal variant as rs1887428 (PPI = 0.999) at the *JAK2* promoter, but summary statistic imputation combined with multivariate fine mapping (SuSIE[69]) prioritised the variant rs1327500 (PPI = 0.663), in a region ~20 kb upstream of *JAK2*, without detectable promoter contacts in ILC3 cells or CD4+ T cells (**Fig. S6C**). However, considering that both rs1887428 and rs1327500 are eQTLs for *JAK2* in blood cells, according to eQTLGen[174], *JAK2* remains a strong candidate in this locus by genetic association.
